# Cognitive functioning in postural orthostatic tachycardia syndrome among different body positions: a prospective pilot study (POTSKog study)

**DOI:** 10.1007/s10286-023-00950-0

**Published:** 2023-06-01

**Authors:** Andrea Maier, Lena Schopen, Joana C. Thiel, Katharina Müller, Bruno Fimm, Jörg B. Schulz

**Affiliations:** 1 Department of Neurology, Medical Faculty RWTH Aachen University, Aachen, Germany; 2 Department of Gynaecology, Medical Faculty RWTH Aachen University, Aachen, Germany; 3 JARA-BRAIN Institute Molecular Neuroscience and Neuroimaging, Jülich Research Center GmbH and RWTH Aachen University, Aachen, Germany

**Keywords:** Postural orthostatic tachycardia syndrome (PoTS), Orthostatic intolerance, Executive function, Hyperadrenergic state, Counter manoeuvre, Leg crossing

## Abstract

**Purpose:**

Approximately 96% of patients with postural orthostatic tachycardia syndrome (PoTS) report cognitive complaints. We investigated whether cognitive function is impaired during sitting and active standing in 30 patients with PoTS compared with 30 healthy controls (HCs) and whether it will improve with the counter manoeuvre of leg crossing.

**Methods:**

In this prospective pilot study, patients with PoTS were compared to HCs matched for age, sex, and educational level. Baseline data included norepinephrine plasma levels, autonomic testing and baseline cognitive function in a seated position [the Montreal Cognitive Assessment, the Leistungsprüfsystem (LPS) subtests 1 and 2, and the Test of Attentional Performance (TAP)]. Cognitive functioning was examined in a randomized order in supine, upright and upright legs crossed position. The primary outcomes were the cognitive test scores between HCs and patients with PoTS at baseline testing, and among the different body positions.

**Results:**

Patients with PoTS had impaired attention (TAP median reaction time) in the seated position and impaired executive functioning (Stroop) while standing compared with HC. Stroop was influenced by position (supine versus upright versus upright legs crossed) only in the PoTS group. Leg crossing did not result in an improvement in executive function. In patients with PoTS, there was a negative correlation of Stroop with norepinephrine plasma levels while standing.

**Conclusion:**

Compared with HCs, PoTS participants showed impaired cognitive attention and executive function in the upright position that did not improve in the legs crossed position. Data provide further evidence for orthostatic cognitive deterioration in patients with PoTS.

**Trial Registration Information:**

The study was registered at ClinicalTrials.gov (NCT03681080).

## Introduction

Postural orthostatic tachycardia syndrome (PoTS) is one of the most common forms of chronic orthostatic intolerance and especially affects young women [1–3]. It is characterized by a sustained increase in heart rate (HR) of at least 30 beats/min within 10 mins of standing, accompanied by symptoms such as palpitations, dizziness, headaches and presyncope. In addition to these classical orthostatic symptoms, patients also report fatigue and concentration difficulties [4]. PoTS is primarily considered a disease of the peripheral nervous system. However, since up to 96% of patients with PoTS report cognitive difficulties [5, 6], a central origin has also been discussed [7]. Prior studies showed unimpaired cognition in a supine position but impaired attention, memory, cognitive processing speed and executive function in a seated or upright position [3, 5, 8–13]. While most studies investigated cognitive impairment during passive standing (tilt table), active standing can better represent everyday life, and cardiovascular compensation differs compared with passive standing on a tilt table [14]. Large muscles contract during active standing and release pressure on veins in the lower extremities; thus, skeletal muscle pumps improve venous return to the heart [15]. Therefore, leg crossing is an easy and effective way to increase the central blood volume [16]. In PoTS therapy, counter manoeuvres, such as leg crossing, are recommended during acute dizziness [17]. Thus, if cognition is impaired during active standing due to a functional deficit induced by orthostatic stress, then leg crossing might reduce the cognitive impairment. Concerning pathophysiological mechanisms, cerebrovascular mechanisms, such as reduced transcranial perfusion, and an association between sympathetic stress (“hyperarousal”) and cognitive deterioration in patients with PoTS , which are exacerbated during standing, have been discussed [3, 8, 11, 18].

Two key questions were investigated in this study. First, does an upright position (sitting, active standing) impair cognitive function in patients with PoTS compared with healthy controls (HCs), and if so, which functions are affected? Second, does leg crossing improve these cognitive deficits?

## Methods

### Patients and study design

**Fig. 1:**
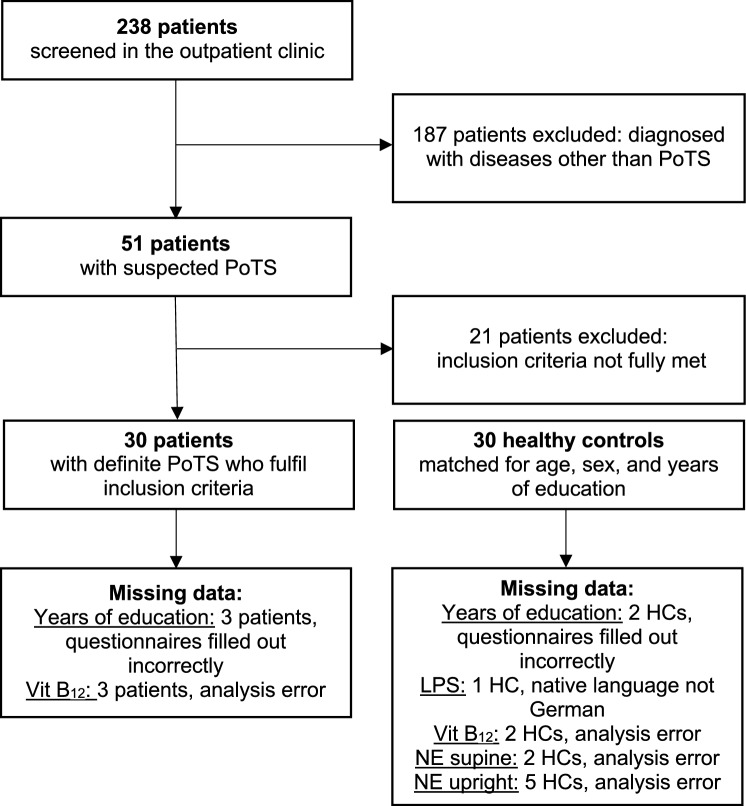
Flow diagram of the study design and inclusion process. Missing data were years of education in three patients and two controls, because they did not fill out questionnaires correctly, and vitamin B_12_ level in three patients and two controls because of analysis error, norepinephrine supine in two controls and upright in five controls because of analysis error and LPS in one control, because her native language was not German

Between May 2017 and March 2021, out of 238 consecutive patients that were examined in the autonomic outpatient clinic of the university clinic, 51 patients were diagnosed with suspected PoTS. Out of these, 30 subjects had definite PoTS, fulfilled the inclusion criteria and were included in the study (Fig. 1). The PoTS group was compared to 30 HCs. HCs were recruited via information flyers online on our homepage, healthy staff and personal contacts (friends, family of our staff and the patients).

The inclusion criterion for patients with PoTS was PoTS diagnosis according to the guidelines of the Canadian Cardiovascular Society [19]. These include a sustained increase in heart rate (HR) of at least 30 beats/min or a sustained HR of 120 beats/min within the first 10 min during standing in the absence of orthostatic hypotension, accompanied by the presence of orthostatic symptoms for at least 3 months. Other causes of tachycardia, such as hypovolemia, cardiac diseases, hyperthyroidism, acute illness, severe deconditioning, infections or use of cardioactive drugs [20, 21] were excluded during the clinical examination process. For HCs, additional exclusion criteria were antihypertensive medications, symptoms of dizziness, a history of syncope, neurological diseases, dementia, known iron deficiency and psychiatric disorders. The HC group was matched for age, gender and years of school education.

### Baseline autonomic examination

The examinations were performed on two different days: on day one, tilt table testing with continuous blood pressure measurement and a medical examination were performed, and on day two, blood sampling and cognitive testing were performed. Baseline cardiovascular autonomic and laboratory testing [norepinephrine (NE) values while supine and standing] were performed in a standardized manner in the morning, without medication, smoking or caffeine intake for at least 12 h. The standing time was 10 min on the tilt table at 70 degrees and blood pressure (BP) and HR changes were continuously measured using Finometer MIDI (Finapres Medical Systems B.V., Enschede, Netherlands) or fan 4.1.0 (BioSign GmbH, Ottenhofen, Germany) [22]. We calculated maximum/mean systolic and diastolic BP and HR changes by the difference in the mean BP or HR in the supine position and the minimum/mean BP or maximum/mean HR during the first 10 min, while being tilted. NE plasma levels were measured after at least 30 mins of rest (NE supine) and after 10 min of active standing (NE upright). Vitamin B_12_ levels were analyzed using the supine blood sample in all participants, to rule out vitamin B_12_ deficiency as potential cause of cognitive decline [23, 24].

### Neuropsychological assessment and questionnaires

There was a break of around 15 min between blood sampling and neuropsychological assessment. Baseline data (cognition baseline) were recorded in a seated position and included the Montreal Cognitive Assessment (MoCA) [25] to exclude dementia, the Leistungsprüfsystem (LPS) subtests 1 and 2 [26] to assess verbal intelligence and the Test of Attentional Performance (TAP), alertness section, to assess attentional deficits [27]. The duration of the cognition baseline testing was approximately 20 min. Self-assessment questionnaires for anxiety [Beck Anxiety Inventory (BAI)] [28] and depressive symptoms [Beck Depression Inventory-II (BDI-II)] [29, 30] were completed by all participants to assess symptoms that might influence cognition.

**Fig. 2:**
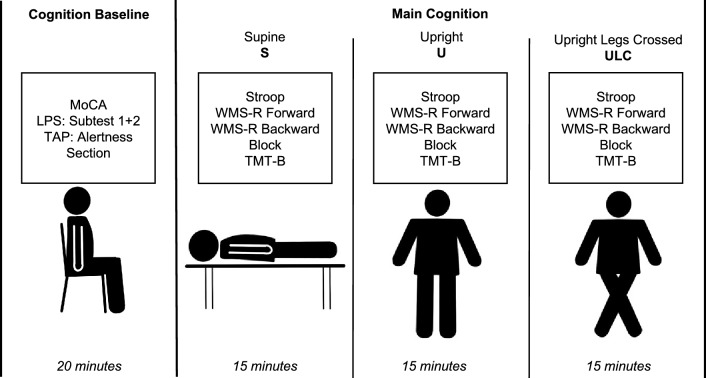
Study protocol. Montreal Cognitive Assessment (MoCA), Leistungsprüfsystem (LPS), Test of Attentional Performance (TAP Test), Wechsler-Memory Scale (WMS-R), Corsi block-tapping task (Block), Trail Making Test B (TMT-B)

The following tests were used to record position-dependent cognition: the Stroop Colour and Word Test (Stroop) [31], a short, easy to learn and well-established test for executive function, which was also used in previous studies to assess the executive function in patients with PoTS [3, 8, 9], the Wechsler Memory Scale-Revised (WMS-R) forward and backward [32], providing global values for general short- and long-term memory [33]—it is one of the most widely used tests for evaluation of memory function in adults [34], the Corsi block-tapping task (Block) [35], a simple yet powerful test [36] that is effectively used to record visual spatial short-term memory [35], and the Trail Making Test subtest B (TMT-B) [37], used as a well-established test to assess cognitive flexibility [38]. It was already used in patients with PoTS in a prior study [3]. These tests were performed in three different positions: in a supine position (S), an upright position (U), and an upright legs crossed position (ULC). For ULC, participants were asked to cross one leg in front of the other and press them together (see Fig. 2) and told not to change their position during the subsequent cognitive tests. Each run was performed in a different body position (S, U, ULC) resulting in a total of six different orders of the positions: S-U-ULC, S-ULC-U, U-S-ULC, U-ULC-S, ULC-S-U and ULC-U-S. Participants and HC were in blocks assigned to one of the six orders of the positions, dependent on the time of inclusion in the study. Thus, five patients and five HCs each completed the examination in the same order of the positions. The tests during one run were always performed in the same order: (1) Stroop, (2) WMS-R forward, (3) WMS-R backward, (4) Block and (5) TMT-B. The tasks were presented to the participants on a music holder at chest level. Between the different positions, participants were instructed to lie down for 5 min to stabilize circulation. The duration of the cognitive testing during one position took approximately 15 min.

### Statistical analysis

Statistical analysis was performed using SPSS (IBM SPSS Statistics, version 27.0) and R (R-Core Team, version 4.1.2). For LPS, TAP, Stroop, WMS-R and TMT-B, raw scores were converted to T-scores based on population norms for age, gender and educational level. After conversion to T-score population norms were 50 ± 10. To capture the cognitive change between the three positions, the difference in scores from supine to upright (S-U), supine to upright legs crossed (S-ULC), and upright to upright legs crossed (U-ULC) were calculated. Clinical characteristics and cognition baseline results for normally distributed data were compared between groups (PoTS versus HC) using two-sample *t* tests. BAI and BDI-II scores and non-normally distributed data were assessed using non-parametric Mann‒Whitney *U* tests. Position-dependent cognitive outcomes were calculated using a mixed ANOVA [39] with group (PoTS versus HC) as the between-factor and position (S versus U versus ULC) as the within-factor. Due to variance inhomogeneity, for Stroop and WMS-R backward, a robust mixed ANOVA was calculated using the R package “WRS2” [40]. Associations between the variables were examined using Pearson product moment correlation for metric data and Kendall’s tau-b for ordinal data. Data are presented as the mean (M) ± standard deviation (SD) for normally distributed data and median (md) and interquartile range (IQR) for non-normally distributed data. *p* Values < 0.05 were classified as statistically significant.

## Results

The demographic characteristics of all participants are presented in Table 1. The PoTS group was compared to 30 HCs matched for age, sex and educational level [PoTS 33 (25.75–40) years, HC 31 (25–38) years, 25 female and 5 male participants in each group]. No significant difference was found between HC and patients with PoTS concerning vitamin B_12_ levels as possible influence on cognition. Fourteen patients with PoTS fulfilled the criteria for hypermobile joint syndromes [one patient with classical Ehlers‒Danlos syndrome (EDS), one with Marfan syndrome, nine with hypermobile EDS (hEDS), three with hypermobility spectrum disorder], three patients had a prior diagnosis of mast cell activation syndrome (MCAS), four patients had suspected MCAS and two patients had a prior diagnosis of depression.

**Table 1:** Demographics, clinical data and autonomic evaluation of patients with PoTS compared with healthy controls

	PoTS	HC	*p* value
	(M ± SD) or md (IQR) [range] *N* = 30	(M ± SD) or md (IQR) [range] *N* = 30	
Demographics			
Age (years)	33 (25.75–40) [20–53]	31 (25–38) [22–59]	0.749
Sex			
Female	25	25	
Male	5	5	
Years of education	13.00 (11.00–13.00)	13.00 (12.00–13.00)	0.676
Autonomic examination			
Vitamin B_12_, ng/l	496.00 (379.00–699.00)	392.50 (296.50–497.50)	0.068
NE supine (ng/l)	309.00 (175.00–405.75)	173.00 (127.00–283.50)	0.006**
NE upright (ng/l)	648.50 (520.00–1053.50)	408.00 (287.50–497.50)	< 0.001***
NE rise (ng/l)	401.50 (312.75–639.25)	223.00 (119.00–268.50)	< 0.001 ***
SBP supine (mmHg)	124.06 ± 15.42	119.65 ± 17.03	0.297
SBP upright (mmHg)	126.89 ± 17.57	121.23 ± 15.68	0.193
DBP supine (mmHg)	72.01 ± 12.82	69.69 ± 11.31	0.459
DBP upright (mmHg)	84.65 ± 15.64	77.20 ± 13.96	0.056
HR supine (bpm)	77.65 ± 13.81	61.81 ± 9.39	< 0.001***
HR upright (bpm)	108.31 ± 18.72	69.99 ± 10.06	< 0.001***
Max. HR (bpm)	131.01 (123.28–147.33)	85.41 (76.73–90.27)	< 0.001***
Max. HR rise (bpm)	54.01 (46.28–61.16)	22.70 (17.33–26.38)	< 0.001***

Bold values indicate significant differences. *N* = 30 for PoTS and HC for all parameters except years of education: *n*_POTS_ = 27, *n*_HC_ = 28, vitamin B_12_: *n*_POTS_ = 27, *n*_HC_ = 28, NE supine *n*_HC_ = 28, NE upright *n*_HC_ = 25. For age the range is indicated in square brackets. Data are presented as the mean ± SD for normally distributed data and median (IQR) [Range] for non-normally distributed data

*NE* norepinephrine plasma levels, *SBP* systolic blood pressure, *DBP* diastolic blood pressure, *HR* heart rate, *bpm* beats/minute, *max* maximal

*p < 0.05

**p < 0.01

***p < 0.001

### Autonomic examination

**Fig. 3:**
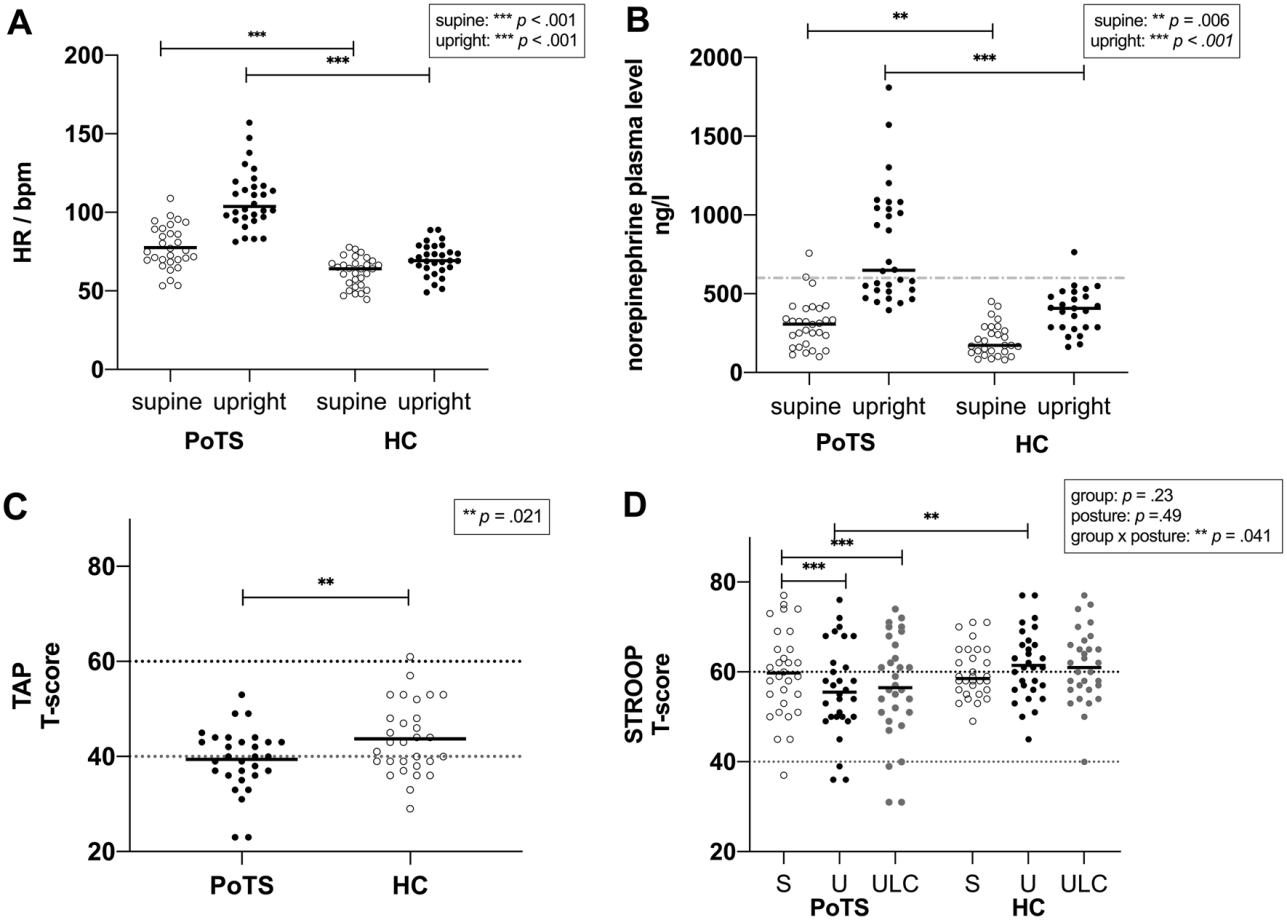
Heart rate (HR) in beats/minute (bpm) (**A**) and norepinephrine (NE) (**B**) change from supine to upright, Test of Attentional Performance (TAP) median results (**C**) and Stroop (**D**) in PoTS compared with healthy controls (HCs) in supine (S), upright (U), and upright legs crossed (ULC) posture. *N* = 30 for patients with PoTS and healthy controls in heart rate, TAP median and Stroop and for patients with PoTS in norepinephrine level. For controls supine, *n* = 28 and for controls upright *n* = 25. Black horizontal lines indicate the means for each group. The dotted grey line in panel B indicates a pathological value > 600 ng/l. The dotted lines in panels C and D are ± 1 SD of the mean of the population within the average range, with T-scores < 40, representing a performance below average. *p < 0.05. **p < 0.01. ***p < .001

As expected, the maximum HR rise was higher in patients with PoTS than in HCs [PoTS 54.01 (46.28–61.16) bpm, HC 22.70 (17.33–36.48) bpm, *p* < 0.001], and the absolute values of HR supine (PoTS 77.65 ± 13.81 bpm, HC 61.81 ± 9.39 bpm, *p* < 0.001), HR upright (PoTS 108.31 ± 18.72 bpm, HC 69.99 ± 10.06 bpm, *p* < 0.001) and the maximum HR during the tilt table [PoTS 131.01 (123.28–147.33) bpm, HC 85.41 (76.73–90.27) bpm, *p* < 0.001] were higher in patients with PoTS (see Table 1 and Fig. 3A). Both the supine [PoTS 309.00 (175.00–405.75) ng/l, HC 173.00 (127.00–283.50) ng/l, *p* = 0.006] and upright [PoTS 648.50 (520.00–1053.00) ng/l, HC 408.00 (287.50–497.50) ng/l, *p* < 0.001] NE levels were higher in the PoTS group, with 53% of patients with PoTS having upright NE plasma levels higher than 600 ng/l, indicating a hyperadrenergic state (see Fig. 3B).

### Neuropsychological assessment and questionnaires

One HC did not take part in the LPS because his native language was not German. The total scores of depression and anxiety symptoms were higher in the PoTS group [BDI II (*N*_PoTS_ = 30, *N*_HC_ = 30): *U* = 164.5, *z* = −4.23, *p* < 0.001, *r* = 0.55, BAI (*N*_PoTS_ = 30, *N*_HC_ = 30): *U* = 92, *z* = −5.32, *p* < 0.001, *r* = 0.69] (Table 2). NE rise correlated moderately positively with BAI (*t*_*b*_ (53) = 0.49, *p* < 0.001) and mildly positively with BDI-II (*t*_*b*_ (53) = 0.28, *p* < 0.003).

**Table 2:** Results of cognitive testing for patients with PoTS compared with healthy controls

	PoTS	HC	*p* value
	(M ± SD) or md (IQR) *N* = 30	(M ± SD) or md (IQR) *N* = 30	
Questionnaires and cognition baseline			
MoCa	29.00 (27.00–30.00)	29.00 (28.00–30.00)	0.680
LPS	50.00 (45.00–55.00)	50.00 (50.00–55.00)	0.338
TAP median	39.37 ± 6.65	43.73 ± 7.56	0.021*
BDI-II	12.83 ± 8.72	5.10 ± 6.03	< 0.001***
BAI	21.23 ± 12.28	3.17 ± 4.59	< 0.001***
Position-dependent cognitive tests			
Stroop S	59.77 ± 9.78	59.87 ± 5.66	0.961
Stroop U	56.07 ± 10.24	61.47 ± 7.73	0.025*
Stroop ULC	56.60 ± 11.50	61.27 ± 8.03	0.074
WMS-R forward S	49.50 (46.00–63.75)	50.00 (43.75–57.00)	0.283
WMS-R forward U	53.50 ± 13.89	49.63 ± 10.76	0.233
WMS-R forward ULC	52.33 ± 12.65	50.00 ± 10.28	0.436
WMS-R backward S	50.00 (44.00–53.00)	48.00 (44.00–51.25)	0.655
WMS-R backward U	48.37 ± 12.92	49.63 ± 10.11	0.674
WMS-R backward ULC	48.10 ± 13.52	47.67 ± 8.19	0.881
Block S	6.00 (5.00–6.00)	5.00 (5.00–6.00)	0.240
Block U	6.00 (5.00–6.00)	5.00 (5.00–6.00)	0.241
Block ULC	5.00 (5.00–6.25)	6.00 (5.00–6.00)	0.572
TMT-B S	45.50 (40.75–50.00)	46.00 (44.00–48.00)	0.863
TMT-B U	45.00 ± 11.90	46.70 ± 5.90	0.893
TMT-B ULC	45.67 ± 7.06	46.30 ± 6.05	0.711
Differences between positions for Stroop			
U–S	−3.70 ± 7.47	1.60 ± 6.65	0.005**
ULC–S	−3.17 ± 6.60	1.40 ± 5.48	0.005**
ULC–U	0.53 ± 4.60	−0.20 ± 5.17	0.564

Bold values indicate significant data. Data are presented as the mean ± SD for normally distributed data and median (IQR) for non-normally distributed data. For the differences, the total scores were subtracted between the two positions

*MoCA* Montreal Cognitive Assessment, *LPS* Leistungsprüfsystem, *TAP Test* Test of Attentional Performance, *BAI* Becks Anxiety Inventory, *BDI-II* Beck Depression Inventory II, *WMS-R* Wechsler Memory Scale, *S* supine position, *U* upright position, *ULC* upright legs crossed, *Block* Corsi block-tapping task, *TMT-B* Trail Making Test B

*p < 0.05

**p < 0.01

***p < 0.001

Patients with PoTS had a moderately lower T-score for the TAP median [*t*(58) = −2.38, *p* = 0.02, *d* = 0.62] (Table 2 and Fig. 3C), indicating reduced attention in the PoTS group compared with the HC group. Patients with PoTS scored moderately lower than HCs in the Stroop test while upright [*t*(58) = −2.31, *p* = 0.03, *d* = 0.61], whereas no differences between patients with PoTS and HCs were detected in the supine and upright legs crossed conditions. The robust ANOVA for Stroop showed a significant interaction between position and group [*F*(2, 27.12) = 3.6, *p* < 0.04] only in the PoTS group [*F*(1.67, 48.45) = 5.97, *p* < 0.01], indicating that executive functioning was influenced by position only in patients with PoTS (Fig. 3D). Differences in Stroop scores between positions differed between the groups for supine to upright position [*t*(58) = 2.9, *p* < 0.005,* d* = 0.76], meaning patients with PoTS performed worse while upright compared with supine and worse while legs crossed compared with supine [*t*(58) = 2.92, *p* = 0.005 *d* = 0.77]. There was a negative correlation between Stroop while upright and the rise of NE values [*r*(53) = −0.3, *p* = 0.03], as well as a moderately positive correlation between the TAP median and Stroop while upright [*r*(58) = 0.38, *p* < 0.01]. Investigating possible correlations in the different groups (HC or POTS), there was no correlation between Stroop U and TAP in the PoTS group [*r*(28) = 0.15, *p* = 0.437], but a positive correlation in the control group [*r*(28) = 0.55, *p* = 0.002]. Furthermore, there was a positive correlation for Stroop S and TAP both in the PoTS [*r*(28) = 0.38, *p* = 0.037] and the HC group [*r*(28) = 0.49, *p* = 0.006].

## Discussion

The primary findings of this study were an impairment of attentional performance (TAP) during the seated position and a reduction of executive function (Stroop) while upright in patients with PoTS compared with HCs. Only a few studies have investigated cognitive dysfunction in PoTS, even though many patients with PoTS report cognitive problems in routine clinical examinations [6, 8, 11]. The results of the TAP in seated patients with PoTS compared with HCs provide further evidence that patients with PoTS show selective cognitive impairment of attentional performance, even during minimal orthostatic stress (sitting). This result is especially interesting in the context of the LPS as a measure of general cognitive ability, which showed no difference between patients with PoTS and HCs. Impaired attention in patients with PoTS was found in other studies in a seated position using Ruff 2 + 7 Speed Test [3], WAIS-III digits forward [8], ADHD subscales [18] and CANTAB [41], and also while standing using CogState [8, 9] and TAP subtest for sustained attention [11]. In contrast, recent research found no differences in tonic alertness using the TAP in supine and passive upright positions; however, the sample size was small (PoTS *n* = 8, only neuropathic PoTS) [42]. Patients scored worse than HCs for Stroop in the upright position and deteriorated from supine to the upright (upright and upright legs crossed) positions. In the supine position, where orthostatic stress is reduced to a minimum, no differences in cognitive tests were detected between PoTS and HCs. This validates the hypothesis that orthostatic stress itself impairs executive function in patients with PoTS. These findings are in line with results found in previous research: describing normal executive function in the supine position but an impairment during active standing [9] and in the seated position using Stroop and Trail Making Test B [3]. In line with previous results [9], our results show a moderately positive correlation between impaired attention (TAP) and executive functioning (Stroop). There was a positive correlation between Stroop U and TAP in the HC group, but not in the PoTS group. As Stroop and TAP both require executive control [43], we would have expected the tests to correlate as seen in the HC group, if standing did not have any impact on executive control. On the other hand, for Stroop S (supine) we found a positive correlation with TAP in both the PoTS and the HC group. In recent research, “sustained attention” was tested with the TAP in the supine position and at 60° head-up tilt during, before, and after water ingestion. There was more cognitive impairment during head-up tilt in patients with PoTS (more omissions in the TAP), which also indicates a decrease in working memory [11]. A positive effect on working memory was shown previously using intake of water to reduce orthostatic symptoms [11, 42]. It must be mentioned that in their study, cognitive performance was tested during passive tilt testing, whereas in our study, patients performed active standing, which pre-activates the leg muscle pump as described above. Thus, all these data support the hypothesis that cognitive impairment in PoTS is not a global problem caused by the disease itself, but a functional deficit induced by orthostatic stress, which might alter cerebral perfusion or central neurometabolic mechanisms. A second important finding was that leg crossing did not improve executive function in patients with PoTS. Crossing the legs increases the venous return and improves cerebral perfusion, but also reduces the balance compared with standing. A situation with an increasing need to maintain balance might result in impaired cognition [44]. Interestingly, the significant difference that exists between PoTS and HCs in the upright posture is no longer detectable in the ULC posture. While the performance in Stroop worsened on average from U to ULC in the HCs, in patients with PoTS, the performance in Stroop improved during ULC, considering the absolute values. However, the change is very small, and it should be interpreted with caution. For further studies we would suggest reducing orthostatic stress by other methods, e.g., using compression garments that reduce orthostatic symptoms without affecting balance.

Although the effect of NE, not only on orthostatic symptoms such as tachycardia, palpitations and tremor, but also on cognitive dysfunction in patients with PoTS, has been extensively discussed in the literature, there is little research and evidence to date. Thus, in this study, one finding was that NE levels were elevated in both the supine and upright positions, similar to previous research [11], indicating an overactivity of the sympathetic nervous system in patients with PoTS compared with HCs. Moreover, there was a negative correlation between the degree of NE rise and Stroop performance while upright. In our study, we can exclude an effect of stress during the cognitive test on NE release because cognitive testing and NE testing were not performed at the same time, as recorded in another study [11]. An excessive NE rise in the PoTS group might negatively influence cognition, either by the central effects of NE itself or more profound symptoms during standing, as described previously [11]. In contrast, an association between plasma levels of NE and impaired cognition was not found, but their cognitive tests were performed in the seated position [3]. In our sample, TAP median values, which were also tested in a seated position, did not correlate with NE responses.

We observed higher depression and anxiety scores using the BDI-II and BAI for patients with PoTS than for HCs. There was a positive correlation between BAI and BDI-II scores and NE increase. The symptoms listed in the BAI include both psychological and somatic symptoms, such as tremor, palpitation, sleep problems and fatigue, which are very common in PoTS and may be a symptom of the hyperadrenergic state and the disease itself, but not of a depressive or anxiety disorder. Only two of our patients had a prior diagnosis of depression, and none had anxiety. These results show that the scores alone must be interpreted with caution [3, 45], especially in patients with PoTS, because PoTS symptoms can mimic symptoms of depression or anxiety. Patients with higher NE levels might score higher in the BAI due to more somatic symptoms caused by PoTS. As symptoms of PoTS are phenomenologically different from symptoms of panic disorder or anxiety, these diseases must be clinically distinguished [46] to avoid misinterpretation of PoTS as an anxiety disorder.

### Study limitations

A limitation of this study is the small study group; thus, further studies should include more patients to reach good power. Moreover, a selection bias cannot be ruled out because in this centre specializing in autonomic disorders, people with less symptom burden may be under represented. Thus, our results are not representative of the overall PoTS population. Second, cognitive tests of the assessment battery did not cover all aspects of cognition, and the TAP was not performed in the standing position due to practical reasons, so the comparison with other studies is limited. To counteract the potential bias caused by practice effects on the results, we randomized the order of positions in which the participants performed the task (S, U, ULC). Thus, any practice effect that may have occurred would be evenly distributed across all the different orders, minimizing its impact on the overall results. This was done both for the PoTS and control groups to ensure that any differences observed between the two groups were solely due to group affiliation (PoTS versus control) and not due to the influence of practice effects. We cannot exclude that a longer standing time with e.g., higher heart rate, lower blood pressure or reduced intracerebral blood flow might influence cognition as well. Interestingly, we started the runs always with the Stroop tests and this was the only test where we found significant differences between the PoTS and the HC group in the main cognition. Thus, one might assume that not the standing time itself, but the upright posture alone may be the important factor. For further studies it might be interesting to test if the standing time has any influence on cognition itself.

## Conclusions

Our study investigated a broad spectrum of cognitive performance and confirmed that cognitive performance concerning attention and executive function is impaired in patients with PoTS during upright positions, including active standing. Leg crossing does not improve executive function. This might be due to reduced balance that influences cognition. Cognitive impairment in PoTS seems not to be a global problem caused by the disease itself, but a functional deficit induced by orthostatic stress. Further studies with larger sample sizes, inclusion of the TAP in the upright position and a combination of NE measures and assessment of cerebral perfusion should be performed to confirm these results and gain further information concerning the pathophysiological mechanisms.

## Data Availability

Anonymized data and the full trial protocol will be shared by reasonable request from the corresponding author.

## References

[1] Shaw BH, Stiles LE, Bourne K, Green EA, Shibao CA, Okamoto LE, Garland EM, Gamboa A, Diedrich A, Raj V, Sheldon RS, Biaggioni I, Robertson D, Raj SR (2019). The face of postural tachycardia syndrome—insights from a large cross-sectional online community-based survey. J Intern Med.

[2] Arnold AC, Ng J, Raj SR (2018). Postural tachycardia syndrome—diagnosis, physiology, and prognosis. Auton Neurosci.

[3] Arnold AC, Haman K, Garland EM, Raj V, Dupont WD, Biaggioni I, Robertson D, Raj SR (2015). Cognitive dysfunction in postural tachycardia syndrome. Clin Sci (Lond).

[4] McDonald C, Koshi S, Busner L, Kavi L, Newton JL (2014). Postural tachycardia syndrome is associated with significant symptoms and functional impairment predominantly affecting young women: a UK perspective. BMJ Open.

[5] Owens MT, Harbeck-Weber C, Kirsch A, Sim L, Zaccariello M, Homan K, Fischer P (2019). Neurocognitive difficulties among youth with POTS within an intensive pain rehabilitation program. J Pediatr Psychol.

[6] Ross AJ, Medow MS, Rowe PC, Stewart JM (2013). What is brain fog? An evaluation of the symptom in postural tachycardia syndrome. Clin Auton Res.

[7] Blitshteyn S (2022). Is postural orthostatic tachycardia syndrome (POTS) a central nervous system disorder?. J Neurol.

[8] Anderson JW, Lambert EA, Sari CI, Dawood T, Esler MD, Vaddadi G, Lambert GW (2014). Cognitive function, health-related quality of life, and symptoms of depression and anxiety sensitivity are impaired in patients with the postural orthostatic tachycardia syndrome (POTS). Front Physiol.

[9] Miller AJ, Sheehan T, Bourne KM, Feeley M, Arnold AC (2020). Attention and executive function are impaired during active standing in postural tachycardia syndrome. Auton Neurosci.

[10] Ocon AJ, Messer ZR, Medow MS, Stewart JM (2012). Increasing orthostatic stress impairs neurocognitive functioning in chronic fatigue syndrome with postural tachycardia syndrome. Clin Sci (Lond).

[11] Rodriguez B, Hochstrasser A, Eugster PJ, Grouzmann E, Müri RM, Z'Graggen WJ (2022). Brain fog in neuropathic postural tachycardia syndrome may be associated with autonomic hyperarousal and improves after water drinking. Front Neurosci.

[12] Wells R, Paterson F, Bacchi S, Page A, Baumert M, Lau DH (2020). Brain fog in postural tachycardia syndrome: an objective cerebral blood flow and neurocognitive analysis. J Arrhythm.

[13] Stewart JM, Medow MS, Messer ZR, Baugham IL, Terilli C, Ocon AJ (2012). Postural neurocognitive and neuronal activated cerebral blood flow deficits in young chronic fatigue syndrome patients with postural tachycardia syndrome. Am J Physiol Heart Circ Physiol.

[14] Plash WB, Diedrich A, Biaggioni I, Garland EM, Paranjape SY, Black BK, Dupont WD, Raj SR (2013). Diagnosing postural tachycardia syndrome: comparison of tilt testing compared with standing haemodynamics. Clin Sci (Lond).

[15] Rowell LB (1993). Human cardiovascular control.

[16] Wieling W, van Dijk N, Thijs RD, de Lange FJ, Krediet CT, Halliwill JR (2015). Physical countermeasures to increase orthostatic tolerance. J Intern Med.

[17] Fu Q, Levine BD (2018). Exercise and non-pharmacological treatment of POTS. Auton Neurosci.

[18] Raj V, Haman KL, Raj SR, Byrne D, Blakely RD, Biaggioni I, Robertson D, Shelton RC (2009). Psychiatric profile and attention deficits in postural tachycardia syndrome. J Neurol Neurosurg Psychiatry.

[19] Raj SR, Guzman JC, Harvey P, Richer L, Schondorf R, Seifer C, Thibodeau-Jarry N, Sheldon RS (2020). Canadian cardiovascular society position statement on postural orthostatic tachycardia syndrome (POTS) and related disorders of chronic orthostatic intolerance. Can J Cardiol.

[20] Freeman R, Wieling W, Axelrod FB, Benditt DG, Benarroch E, Biaggioni I, Cheshire WP, Chelimsky T, Cortelli P, Gibbons CH, Goldstein DS, Hainsworth R, Hilz MJ, Jacob G, Kaufmann H, Jordan J, Lipsitz LA, Levine BD, Low PA, Mathias C, Raj SR, Robertson D, Sandroni P, Schatz IJ, Schondorf R, Stewart JM, Van Dijk JG (2011). Consensus statement on the definition of orthostatic hypotension, neurally mediated syncope and the postural tachycardia syndrome. Auton Neurosci.

[21] Vernino S, Bourne KM, Stiles LE, Grubb BP, Fedorowski A, Stewart JM, Arnold AC, Pace LA, Axelsson J, Boris JR, Moak JP, Goodman BP, Chémali KR, Chung TH, Goldstein DS, Diedrich A, Miglis MG, Cortez MM, Miller AJ, Freeman R, Biaggioni I, Rowe PC, Sheldon RS, Shibao CA, Systrom DM, Cook GA, Doherty TA, Abdallah HI, Darbari A, Raj SR (2021). Postural orthostatic tachycardia syndrome (POTS): state of the science and clinical care from a 2019 national institutes of health expert consensus meeting—part 1. Auton Neurosci.

[22] Billig SCI, Schauermann JC, Rolke R, Katona I, Schulz JB, Maier A (2020). Quantitative sensory testing predicts histological small fiber neuropathy in postural tachycardia syndrome. Neurol Clin Pract.

[23] Bailey RL, Jun S, Murphy L, Green R, Gahche JJ, Dwyer JT, Potischman N, McCabe GP, Miller JW (2020). High folic acid or folate combined with low vitamin B-12 status: potential but inconsistent association with cognitive function in a nationally representative cross-sectional sample of US older adults participating in the NHANES. Am J Clin Nutr.

[24] Langan RC, Goodbred AJ (2017). Vitamin B12 deficiency: recognition and management. Am Fam Physician.

[25] Nasreddine ZS, Phillips NA, Bédirian V, Charbonneau S, Whitehead V, Collin I, Cummings JL, Chertkow H (2005). The Montreal cognitive assessment, MoCA: a brief screening tool for mild cognitive impairment. J Am Geriatr Soc.

[26] Horn W (1983). Leistungsprüfsystem: LPS.

[27] Zimmermann P, Fimm B (1992). Testbatterie zur Aufmerksamkeitsprüfung: (TAP).

[28] Beck AT, Epstein N, Brown G, Steer RA (1988). An inventory for measuring clinical anxiety: psychometric properties. J Consult Clin Psychol.

[29] Beck AT, Steer RA, Brown G (1996) Beck Depression Inventory–II. Psychological Assessment, San Antonio, TX

[30] Wang YP, Gorenstein C (2013). Psychometric properties of the beck depression inventory-II: a comprehensive review. Braz J Psychiatry.

[31] Jensen AR, Rohwer WD (1966). The stroop color-word test: a review. Acta Psychol.

[32] Jones G, Macken B (2015). Questioning short-term memory and its measurement: why digit span measures long-term associative learning. Cognition.

[33] Marazziti D, Catena Dell'osso M, Conversano C, Consoli G, Vivarelli L, Mungai F, Di Nasso E, Golia F (2008). Executive function abnormalities in pathological gamblers. Clin Pract Epidemiol Ment Health.

[34] Hassaan SH, Khalifa H, Darwish AM (2021). Effects of extended abstinence on cognitive functions in tramadol-dependent patients: a cohort study. Neuropsychopharmacol Rep.

[35] Kessels RP, Van Zandvoort MJ, Postma A, Kappelle LJ, De Haan EH (2000). The corsi block-tapping task: standardization and normative data. Appl Neuropsychol.

[36] Siddi S, Preti A, Lara E, Brébion G, Vila R, Iglesias M, Cuevas-Esteban J, López-Carrilero R, Butjosa A, Haro JM (2020). Comparison of the touch-screen and traditional versions of the Corsi block-tapping test in patients with psychosis and healthy controls. BMC Psychiatry.

[37] Arnett JA, Labovitz S (1995). Effect of physical layout in performance of the trail making test. Psychol Assess.

[38] Kortte KB, Horner MD, Windham WK (2002). The trail making test, part B: cognitive flexibility or ability to maintain set?. Appl Neuropsychol.

[39] Cleophas TJ, Zwinderman AH, van Ouwerkerk B (2012). Clinical research: a novel approach to the analysis of repeated measures. Am J Ther.

[40] Mair P, Wilcox R (2020). Robust statistical methods in R using the WRS2 package. Behav Res Methods.

[41] Wells R, Malik V, Brooks AG, Linz D, Elliott AD, Sanders P, Page A, Baumert M, Lau DH (2020). Cerebral blood flow and cognitive performance in postural tachycardia syndrome: insights from sustained cognitive stress test. J Am Heart Assoc.

[42] Rodriguez B, Zimmermann R, Gutbrod K, Heinemann D, Z'Graggen WJ (2019). Orthostatic cognitive dysfunction in postural tachycardia syndrome after rapid water drinking. Front Neurosci.

[43] Miyake A, Friedman NP, Emerson MJ, Witzki AH, Howerter A, Wager TD (2000). The unity and diversity of executive functions and their contributions to complex “Frontal Lobe” tasks: a latent variable analysis. Cogn Psychol.

[44] Bondar A (2003) Balance and cognition: Resource allocation and its control in young and older adults. In: Doctoral Dissertation. Freie Universität Berlin

[45] Raj SR (2013). Postural tachycardia syndrome (POTS). Circulation.

[46] Khurana RK (2006). Experimental induction of panic-like symptoms in patients with postural tachycardia syndrome. Clin Auton Res.

